# Differential Bone Loss in Mouse Models of Colon Cancer Cachexia

**DOI:** 10.3389/fphys.2016.00679

**Published:** 2017-01-11

**Authors:** Andrea Bonetto, Joshua K. Kays, Valorie A. Parker, Ryan R. Matthews, Rafael Barreto, Melissa J. Puppa, Kyung S. Kang, James A. Carson, Theresa A. Guise, Khalid S. Mohammad, Alexander G. Robling, Marion E. Couch, Leonidas G. Koniaris, Teresa A. Zimmers

**Affiliations:** ^1^Department of Surgery, Indiana University School of MedicineIndianapolis, IN, USA; ^2^Department of Otolaryngology, Head and Neck Surgery, Indiana University School of MedicineIndianapolis, IN, USA; ^3^Simon Cancer Center, Indiana University School of MedicineIndianapolis, IN, USA; ^4^Indiana University-Purdue University at Indianapolis, Center for Cachexia Research, Innovation and Therapy, Indiana University School of MedicineIndianapolis, IN, USA; ^5^Department of Exercise Science, University of South CarolinaColumbia, SC, USA; ^6^Department of Anatomy and Cell Biology, Indiana University School of MedicineIndianapolis, IN, USA; ^7^Department of Medicine, Indiana University School of MedicineIndianapolis, IN, USA

**Keywords:** muscle wasting, cachexia, bone loss, osteopenia, osteoporosis, colon cancer

## Abstract

Cachexia is a distinctive feature of colorectal cancer associated with body weight loss and progressive muscle wasting. Several mechanisms responsible for muscle and fat wasting have been identified, however it is not known whether the physiologic and molecular crosstalk between muscle and bone tissue may also contribute to the cachectic phenotype in cancer patients. The purpose of this study was to clarify whether tumor growth associates with bone loss using several experimental models of colorectal cancer cachexia, namely C26, HT-29, and Apc^Min/+^. The effects of cachexia on bone structure and strength were evaluated with dual energy X-ray absorptiometry (DXA), micro computed tomography (μCT), and three-point bending test. We found that all models showed tumor growth consistent with severe cachexia. While muscle wasting in C26 hosts was accompanied by moderate bone depletion, no loss of bone strength was observed. However, HT-29 tumor bearing mice showed bone abnormalities including significant reductions in whole-body bone mineral density (BMD), bone mineral content (BMC), femoral trabecular bone volume fraction (BV/TV), trabecular number (Tb.N), and trabecular thickness (Tb.Th), but no declines in strength. Similarly, cachexia in the Apc^Min/+^ mice was associated with significant decreases in BMD, BMC, BV/TV, Tb.N, and Tb.Th as well as decreased strength. Our data suggest that colorectal cancer is associated with muscle wasting and may be accompanied by bone loss dependent upon tumor type, burden, stage and duration of the disease. It is clear that preserving muscle mass promotes survival in cancer cachexia. Future studies will determine whether strategies aimed at preventing bone loss can also improve outcomes and survival in colorectal cancer cachexia.

## Introduction

Colorectal cancer represents the third most common cancer in the United States and worldwide (Siegel et al., 2016) and is associated with the development of cachexia in up to 30% of the cases. Cachexia, defined as loss of body weight and depletion of muscle mass (i.e., sarcopenia), with or without loss of fat tissue (Fearon et al., 2011), represents a devastating complication of cancer (Costelli and Baccino, 2003; Tisdale, 2009; Fearon et al., 2012). It has been estimated that up to 80% of cancer patients will develop cachexia over the course of their disease (Haehling and Anker, 2010). The development of cachexia often results in worsened quality of life, decreased tolerance to radio- and chemotherapy, and overall reduced survival. Indeed, it is estimated that cachexia is responsible for 25–30% of all cancer-related deaths (Tisdale, 2009; Muscaritoli et al., 2010). In cancer patients, cachexia is generally diagnosed in association with unintentional weight loss of at least 5% of initial weight and is normally accompanied by muscle weakness, fatigue, anorexia, changes in body composition (including lean and fat mass), increased inflammatory state, anemia and low levels of serum albumin. Of note, it has been shown that body and muscle weight loss positively correlate with enhanced mortality (Evans et al., 2008; Fearon et al., 2011).

Interestingly, while cancer patients have an increased risk of bone loss and osteoporosis, as often shown in patients affected with lung cancer (Fearon, 1992) or in those undergoing radio- or chemotherapy treatments (Mcdonald et al., 2016; Monroy-Cisneros et al., 2016), this feature represents a largely unexplored aspect of cachexia research. A growing body of evidence has led to the identification of molecular mechanisms and signaling pathways associated with muscle and fat loss in cancer. Whether the same pathways also interfere with the homeostasis of bone tissue is not completely clear. Along this line, it has been proposed that similar mechanisms associated with muscle wasting may also play a fundamental role in promoting cancer-associated bone loss, thus leading to the hypothesis that muscle and bone are regulated in tandem in cachexia (Kandarian, 2008). In more recent years, several reports suggested that osteoporosis as well as bone metabolic dysfunction and the decay of bone tissue may represent one of the peculiarities of cachexia and may participate directly in cachexia development and sustainment (Verschueren et al., 2013; Huo et al., 2015). Further, bone and muscle tissues, besides playing a fundamental role in body growth and movement, have been recently described as endocrine organs (Karsenty and Ferron, 2012; Pedersen and Febbraio, 2012; Laurent et al., 2016). Interestingly, muscle and bone loss have been correlated in human and animal models during exercise, aging, disuse, and inflammatory conditions such as arthritis and cancer (Digirolamo et al., 2013). Moreover, with increasing recognition of the physiologic and molecular crosstalk between muscle and bone (Cianferotti and Brandi, 2014), mediators shown to be associated with the pathogenesis of skeletal muscle and fat loss were reported to affect bone tissue in a similar manner (Choi et al., 2013; Waning et al., 2015).

The goal of this study was to clarify whether tumor growth is associated with the occurrence of bone loss. For this purpose, cachexia was induced in mice using colorectal cancer xenografts (murine C26 and human HT-29) or genetically induced tumors (Apc^Min^) and the effects in terms of tumor-associated loss of bone mass were assessed. In addition, we examined the effects of colorectal cancer growth on bone structure, and mechanical properties were determined by utilizing dual energy X-ray absorptiometry (DXA), micro computed tomography (μCT) and three-point bending test. Ultimately, our findings provide evidence that the development of bone effects might depend upon tumor type, burden, stage and duration of the disease.

## Materials and methods

### Animals

All animal experiments were conducted with the approval of the Institutional Animal Care and Use Committee at Indiana University School of Medicine and were in compliance with the National Institutes of Health Guidelines for Use and care of Laboratory Animals and with the ethical standards laid down in the 1964 Declaration of Helsinki and its later amendments. Eight-week old CD2F1 male mice (Harlan, Indianapolis, IN) were injected intrascapularly (s.c.) with 1 × 10^6^ C26 (Colon-26) adenocarcinoma cells in sterile saline and sacrificed after 14 days, when body weight loss was about 15% of the initial body weight, a condition referred to as severe cachexia (Bonetto et al., 2011). Control mice received an equal volume of saline. Eight-week old athymic nude (Nu/Nu) male mice (Harlan, Indianapolis, IN) were injected subcutaneously between the scapulae with 2 × 10^6^ HT-29 cells and sacrificed after 47 days from tumor inoculation. Control mice received an equal volume of saline. Twelve-week old C57BL6/J-Apc^Min^/J (Apc^Min/+^) male mice (The Jackson Laboratory, Bar Harbor, ME) were maintained in our colony for up to 27 weeks of age. Animals were genotyped upon delivery, according to the protocol provided by The Jackson Laboratory. Mice were sacrificed when muscle weight loss was about 25% of the initial body weight (i.e., the weight recorded at time of delivery at our facility). Age-matched C57BL6/J mice served as controls (The Jackson Laboratory, Bar Harbor, ME). Animals were monitored and weighed daily until the day of sacrifice. At time of sacrifice, all mice displayed evident tumor growth and no animals were excluded from the study. Several tissues were collected, weighed, snap frozen in liquid nitrogen and stored at −80°C for further studies. The tibialis anterior muscle was frozen in liquid nitrogen-cooled isopentane, mounted in OCT and stored for morphological analyses.

### Cell culture

Murine C26 cells were kindly provided by Donna McCarthy (Ohio State University) and cultured in high glucose (4.5 g/L) Dulbecco's Modified Eagle's Medium (DMEM) supplied with 10% fetal bovine serum, 1% glutamine, 1% sodium pyruvate, 1% penicillin/streptomycin. Human HT-29 cells (ATCC, Manassas, VA) were cultured in McCoy's 5a Modified Medium supplied with 10% fetal bovine serum, 1% glutamine, 1% sodium pyruvate, and 1% penicillin/streptomycin. Both cell lines were maintained in a 5% CO_2_, 37°C humidified incubator.

### Dual-energy X-ray absorptiometry (DXA)

Assessment of lean tissue, as well as whole body bone mineral density (BMD) and bone mineral content (BMC) were assessed by means of DXA scanning of frozen carcasses. According to the manufacturer's guidelines, in order to calibrate and validate the apparatus for its performance, a spine phantom was scanned using the Lunar PIXImus densitometer (PIXImus, Fitchburg, WI) before scanning the first carcass. Animal carcasses were placed in a prone position with the limbs outstretched. From the whole-body scans, areal BMD and BMC were calculated for the entire body minus head ROI, and regionally for humerus, femur, and lumbar spine (L5) using the Lunar ROI tools.

### Micro computed tomography (MCT)

After euthanasia, the right femur was dissected from each mouse, fixed for 2 days in 10% neutral buffered formalin, and then transferred into 70% ethanol for μCT scanning on a high-throughput μCT specimen scanner (μCT-35; Scanco Medical AG). The distal 33% of each bone was scanned using the following conditions: 50 kV, 120 mA, 151-ms integration time, 0.5 mm Al filter, and 10-μm voxel resolution (Bouxsein et al., 2010). Three-dimensional morphometric properties of the distal femur cancellous bone were measured as previously described (Niziolek et al., 2011). Briefly, trabecular bone volume fraction (BV/TV; %), trabecular number (Tb.N; 1/mm), and trabecular thickness (Tb.Th; mm) were determined on a 1.5 mm region of the distal femur secondary spongiosa, using an ROI beginning 0.6 mm proximal to the distal growth plate (identified by radiolucency and morphology) and extending proximally for 1.5 mm. The trabecular bone was digitally isolated from the cortical compartment by manually lassoing the trabecular bone every 15 slices, then interpolating the trabecular compartment in intervening slices using the contouring function in the Scanco software. All measurements were calculated automatically using the Scanco software (μCT v6.1).

### Three-point bending test

In order to define the bone mechanical properties in the presence of colorectal cancer, the bones were loaded to failure by three-point bending. Briefly, the left femurs were removed from the carcasses, wrapped in saline soaked gauze, and stored at −20°C. Prior to testing they were rehydrated overnight in 0.9% NaCl at room temperature. Testing was performed on a miniature materials testing machine (Vitrodyne V1000; Liveco, Inc., Burlington, VT, USA), which has a force resolution of 0.05 n. The lower supports were set at the maximal allowable distance for each bone without compromising the test (10.0 mm for the femur). The crosshead speed during testing was 0.2 mm/s, and force-displacement data was collected every 0.01 s. From the data, a force vs. displacement graph was created, and the ultimate force (F_U_; N), stiffness (S; N/mm) and post yield energy to failure (U_PY_; mJ) were calculated as shown in Mcateer et al. (2010).

### Statistical analysis

**Figure 1:**
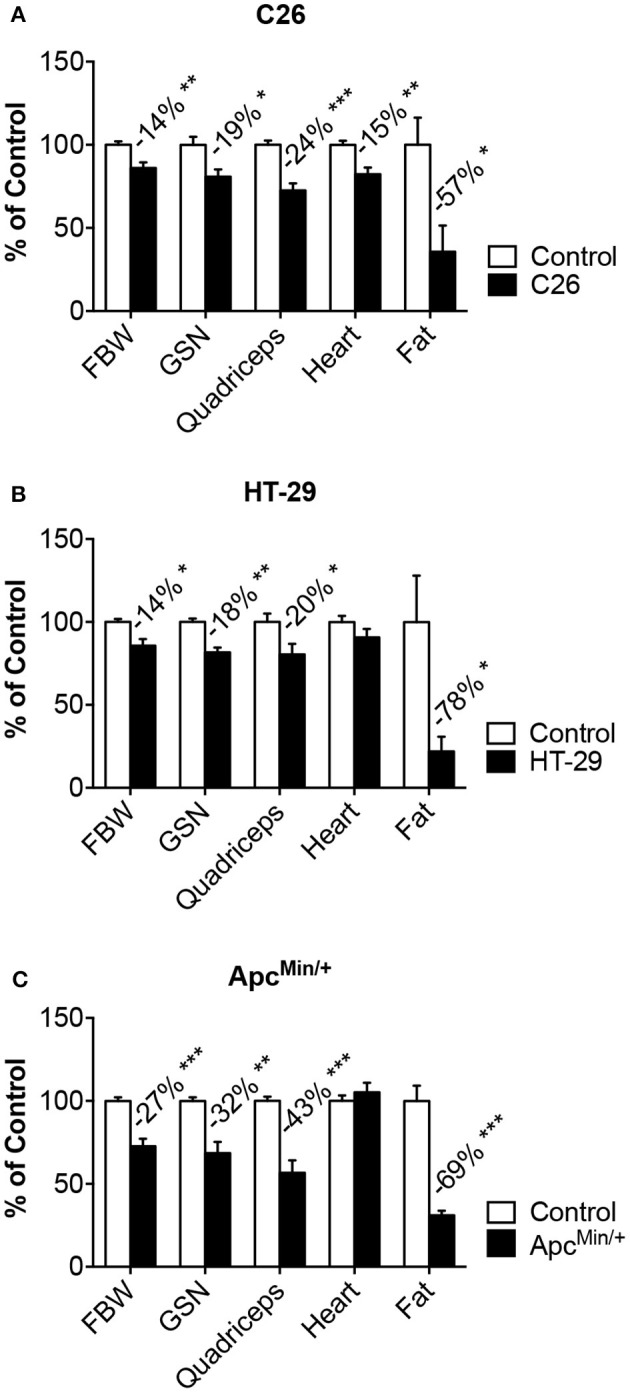
**Models of colorectal cancer show muscle and fat wasting**. Tumor growth was associated with changes in body weight, muscle (gastrocnemius, quadriceps, heart) and fat tissue in mice bearing colorectal cancers. C26 **(A)**, Apc^Min/+^ **(B)**, HT-29 **(C)**. *n* = 4–8. Data (means ± SEM) are expressed as percentage (%) of the respective Control group. Significance of the differences: ^*^p < 0.05, ^**^p < 0.01, ^***^p < 0.001 vs. Control. FBW, tumor-free final body weight; GSN, gastrocnemius.

All results were expressed as means ± SEM. In particular, changes in muscle and fat mass (Figure 1) are presented as percentage (%) of the tissue weights normalized to the initial body weight (IBW). Significance of the differences was evaluated by Student's *t*-test. Difference was considered significant when *p* < 0.05.

## Results

### Colorectal cancer causes body weight loss associated with muscle and fat wasting

In order to investigate whether the growth of colorectal cancer associates with the development of cachexia and bone loss, we took advantage of three different *in vivo* models. In particular, CD2F1 mice injected with the well-characterized C26 murine colorectal cancer cells showed progressive loss of body weight (−14%, *p* < 0.01), accompanied by marked muscle depletion, as suggested by the reduction in skeletal muscle mass (GSN: −16%; Quadriceps: −26% vs. Control) and by the decrease in lean tissue content (*C* = 2.49 ± 0.38 g, C26 = 2.05 ± 0.39 g; −18%, *p* < 0.05), assessed by means of DXA. Consistently, the epididymal fat was also severely depleted in the tumor hosts (−62%, *p* < 0.05) (Figure 1A), while tumor size (0.67 ± 0.29 g) was in line with previous studies using the same experimental model (Bonetto et al., 2011). Of note, tumor growth also caused cardiac muscle atrophy (−17%, *p* < 0.01) (Figure 1A). To the extent of establishing and characterizing new preclinical mouse models of colorectal cancer, we injected the HT-29 human colorectal adenocarcinoma in athymic nude mice. After 47 days from tumor inoculation, the animals showed significantly reduced body weight (−14%; *p* < 0.05 vs. Control) along with depletion of skeletal muscle (GSN: −18%; Quadriceps: −20% vs. Control), overall reduction in lean tissue content (*C* = 6.80 ± 0.63 g, HT-29 = 5.82 ± 0.38 g; −14%, *p* < 0.05) and severe decrease in fat mass (−78%, *p* < 0.05) (Figure 1B). This was also consistent with remarkable tumor growth (2.71 ± 1.37 g). Similarly, at around 27 weeks of age, the Apc^Min/+^ mouse, an extensively studied genetic model of colorectal cancer development, displayed severe body weight loss (−27%, *p* < 0.001), consistent with overall loss of skeletal muscle mass (GSN: −32%; Quadriceps: −43% vs. Control), lean tissue (*C* = 9.67 ± 0.72 g, Apc^Min/+^ = : 5.37 ± 1.11 g; −44%, *p* < 0.001) and adipose tissue (−69%, *p* < 0.001), but no change in heart weight (Figure 1C).

### Bone tissue is differentially affected by colorectal cancer

**Figure 2:**
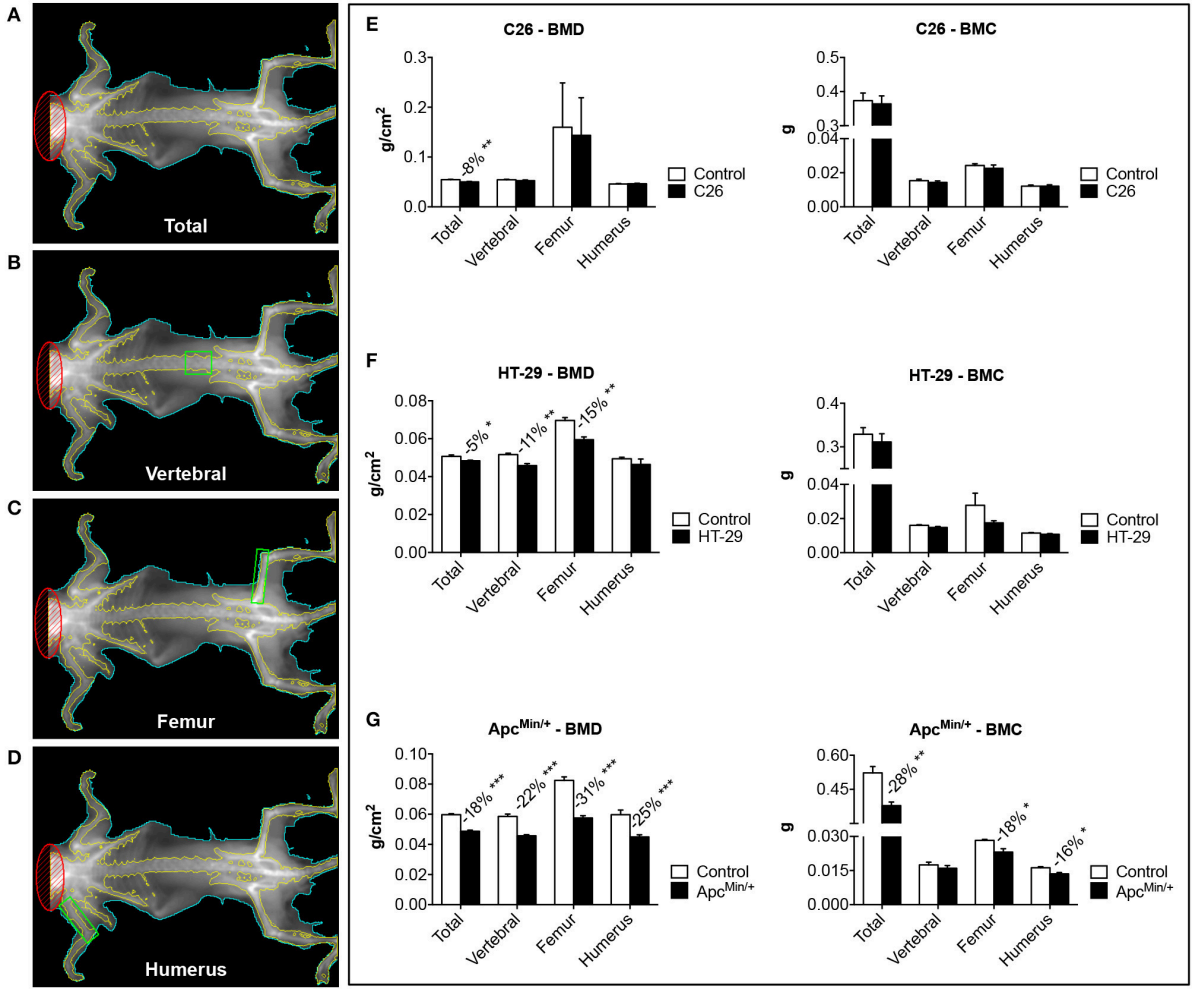
**The development of colorectal cancer is associated with decreases in Bone Mineral Density**. Representative DXA scans of carcasses from colorectal cancer hosts. Total bone mineral density (BMD) and bone mineral content (BMC) measurements of the whole body are shown in **(A)**. Green boxes indicated ROI for the measurement of regional BMD and BMC (**B**: L5 vertebrae; **C**: femur; **D**: humerus). BMD and BMC quantification in C26-bearing animals **(E)**, HT-29 hosts **(F)** and Apc^Min/+^ mice **(G)**. *n* = 4–8. Data (means ± SEM) are expressed as g/cm^2^ (for BMD) or g (for BMC). Significance of the differences: ^*^p < 0.05, ^**^p < 0.01, ^***^p < 0.001 vs. Control.

**Figure 3:**
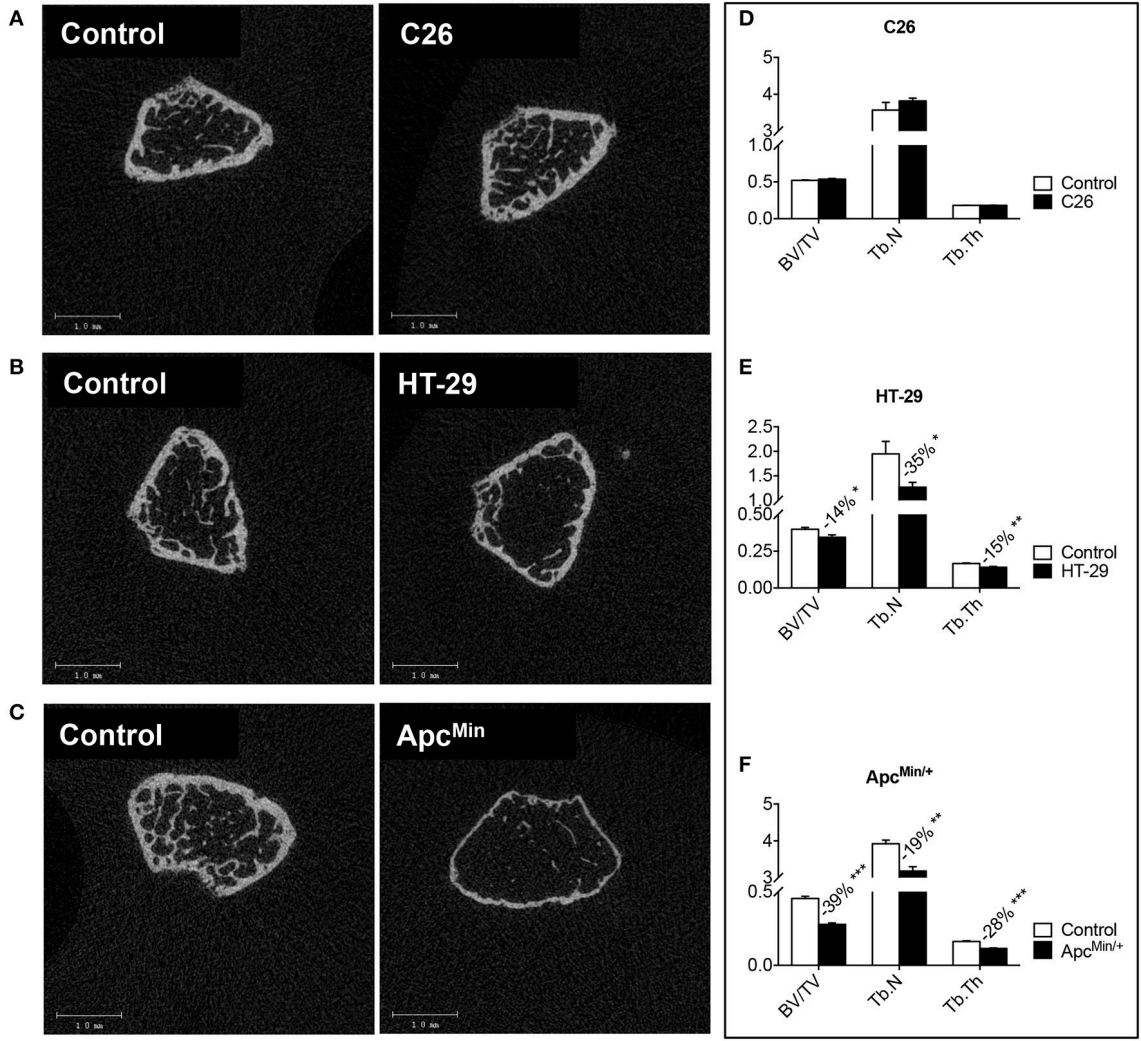
**Colorectal cancer promotes bone degeneration in the Apc^Min/+^ and HT-29-bearing mice, but not in the C26 hosts**. Representative μCT scan images of femur sections from colorectal cancer hosts **(A–C)**. Quantification of bone volume fraction (BV/TV; %), trabecular number (Tb.N; 1/mm), and trabecular thickness (Tb.Th; mm) in the femur of tumor-bearing mice. C26 **(D)**, HT-29 **(E)** and Apc^Min/+^ **(F)**. *n* = 4–8. Data are expressed as means ± SEM. Significance of the differences: ^*^p < 0.05, ^**^p < 0.01, ^***^p < 0.001 vs. Control.

Bone loss in the presence of colorectal cancer was assessed by means of DXA (Figure 2) or μCT scans (Figure 3). Based on the DXA scan quantification, while a moderate loss of whole body bone mineral density (BMD) was observed in the C26 hosts (Figure 2E), the HT-29 hosts showed decreased BMD (−5%, *p* < 0.05), along with depletion of bone tissue at the level of vertebrae (−11%, *p* < 0.01) and femur (−15%, *p* < 0.01) (Figure 2F). Similarly, the Apc^Min/+^ mice displayed an overall severe depletion of bone tissue (−18%, *p* < 0.001), even more exacerbated in the L5 vertebrae (−22%, *p* < 0.001), femur (−31%, *p* < 0.001) and humerus (−25%, *p* < 0.001) (Figure 2G). Interestingly, no significant change in bone mineral content (BMC) was detected in the C26 or HT-29 hosts (Figures 2E,F), while an overall marked bone tissue loss was detected in the Apc^Min/+^ animals (−28%, *p* < 0.01), and more specifically in femur (−18%, *p* < 0.05) and humerus (−16%, *p* < 0.05) (Figure 2G). Consistently, the μCT analysis revealed varying levels of bone loss across our models. In particular, while no significant changes were observed in the C26 model (Figures 3A–D), decreased BV/TV (−14%, *p* < 0.05), Tb.N (−35%, *p* < 0.05) and Tb.Th (−15%, *p* < 0.01) were detected in the bone of mice bearing the HT-29 tumors (Figures 3B–E). Analogously, the Apc^Min/+^ showed reduced BV/TV (−37%, *p* < 0.001), Tb.N (−19%, *p* < 0.01) and Tb.Th (−28%, *p* < 0.001) (Figures 3C–F).

### The Apc^Min/+^ mouse shows reduced bone strength

**Figure 4:**
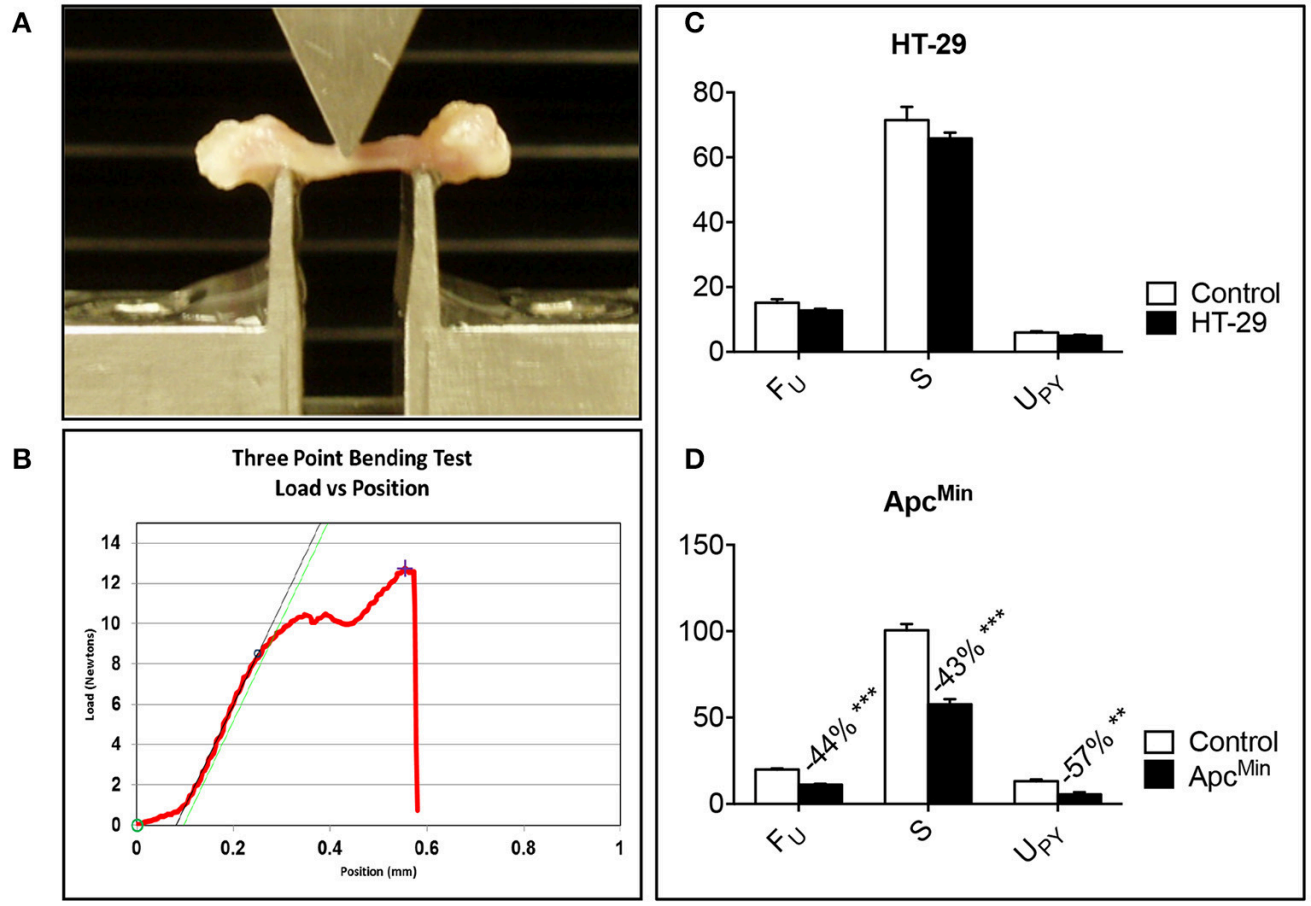
**Bone strength is decreased in the Apc^Min/+^ mouse**. Representative three-point bending test performed on femur from colorectal tumor-bearing mice **(A)**. Representative Force vs. displacement graph, where the peak of the curve represents the ultimate force (F_U_), the slope the stiffness (S), and the area under the curve the energy to failure (U_PY_) **(B)**. HT-29 **(D)**, Apc^Min/+^ **(C)**. *n* = 4–8. Data (N for F_U_; N/mm for S; mJ for U_PY_) are expressed as means ± SEM. Significance of the differences: ^**^p < 0.01, ^***^p < 0.001 vs. Control.

In order to investigate whether changes in bone strength were associated with the development of colorectal cancer *in vivo*, femurs from HT-29 bearers or Apc^Min/+^ mice were subjected to three-point bending mechanical testing. Of note, despite a moderate loss of bone tissue, as shown in Figure 3, no change in bone strength, measured by three-point bending test (Figures 4A,B), was detected in the HT-29 tumor-bearing mice (Figure 4C). Conversely, the Apc^Min/+^ mice showed significant decrease in ultimate force (F_U_; −44%, *p* < 0.001), stiffness (S; −43%, *p* < 0.001), and energy to failure (U_PY_; −57%, *p* < 0.01) when compared to the wild type controls (Figure 4D).

## Discussion

Extensive skeletal muscle wasting, with or without fat depletion, is one of the hallmarks of cancer cachexia (Fearon et al., 2011). Indeed, skeletal muscle loss and weakness are debilitating consequences of several advanced malignancies, which often associate with bone metastases (Waning et al., 2015). While the mechanisms associated with the development of bone metastases have been investigated for quite some time, it is not clear whether the occurrence of tumor-derived muscle wasting also directly affects bone tissue and its mechanical properties. In the present study, we aimed to investigate whether the occurrence of colorectal cancer was also associated with abnormalities in bone structure and mechanical properties. A better understanding of how cancer cachexia impacts the musculoskeletal system requires the generation of proper pre-clinical models for use in mechanistic studies. Indeed, only a handful of mouse models, only partially characterized, are currently in use for the study of cancer cachexia (Mori et al., 1991; Aulino et al., 2010; Benny Klimek et al., 2010). Therefore, we examined new and well-characterized experimental models of colorectal cancer cachexia to determine whether tumor growth is associated with the occurrence of bone pathology.

**Figure 5:**
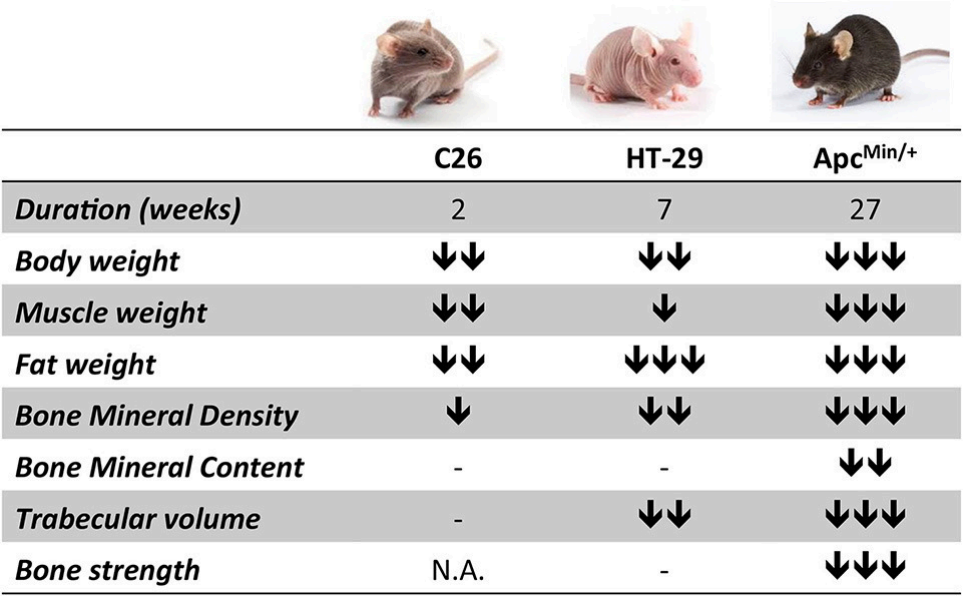
**The degree of bone loss in colorectal cancer cachexia depends upon tumor type, burden and duration of the disease**. The chart summarizes the phenotypic changes observed upon colorectal cancer growth in the three experimental models (C26, HT-29 and Apc^Min/+^). The number of arrows is indicative of the degree of change (mild, moderate, severe).

Here, we show that bone loss, accompanied by aberrations in bone structure and function, is associated with colorectal cancer cachexia by utilizing well-known and new *in vivo* models of colorectal cancer. We examined mice bearing the murine C26 tumor (Bonetto et al., 2009, 2011) or the human HT-29 colorectal adenocarcinoma. We also studied the Apc^Min/+^ mouse (Mehl et al., 2005; Bonetto et al., 2012; White et al., 2012), which carries a heterozygous germ line mutation at codon 850 of the Apc gene responsible for the development of spontaneous colorectal adenomas. We found that all models showed tumor growth consistent with severe cachexia, consistently with muscle loss and fat depletion, although at a different extent across the three models (Figure 5). Interestingly, tumor size also seemed to correlate with the degree of wasting and with the length of the experimental period, whereas the C26 hosts show smaller tumors compared to the HT-29 bearers. In the present work, no assessment of tumor size was performed in the Apc^Min/+^ mice, although it was previously shown that the size of the colorectal polyps mainly correlates with the extent of cachexia (Puppa et al., 2011).

Interestingly, our work is consistent with other studies that have shown a concurrent loss of muscle and bone tissue in murine models of cancer cachexia. Indeed, it was recently reported that animals bearing the Lewis Lung carcinoma present muscle wasting associated with decreased BMD, although alterations of bone strength were not taken into consideration (Choi et al., 2013). Similarly, bone tissue and bone strength were significantly affected in murine models of pancreatic cancer (Greco et al., 2015; Zhang et al., 2015). In the present study, we showed that regardless of the type of tumor, muscle and fat loss were generally accompanied by significantly decreased BMD, while BMC was reduced exclusively in the Apc^Min/+^ animals (Figure 5). Moreover, HT-29 and Apc^Min/+^ tumor hosts also displayed altered bone structure, consistent with decreased BV/TV, Tb.N and Tb.Th. Only the Apc^Min/+^ mouse, characterized by the longest duration of the disease and the most aggressive cachectic phenotype, showed abnormal mechanical properties, as evidenced by the decreased F_U_, S and U_PY_ parameters. Surprisingly, these results are in substantial disagreement with previous reports showing that mutations of the Apc gene, encoding for a protein whose main role is to bind β-catenin, a mediator of the Wnt signaling pathway, are actually associated with increased BMD, both in animal models and in patients with Familial Adenomatous Polyposis (Holmen et al., 2005; Miclea et al., 2010). On the other hand, we cannot exclude that dysfunctions of the gut barrier, as often associated with the development of colorectal cancer in the Apc^Min/+^ mouse model, may not only result into higher inflammation and increased risk of endotoxemia (Puppa et al., 2011), but also in decreased intake of calcium and Vitamin D, required for proper bone formation (Carmeliet et al., 2015; Wesa et al., 2015).

Based on our observations it does appear that the duration of the disease and the extent of muscle loss represent major contributing factors in regulating bone tissue in colorectal cancer. However, our results, generated by analyzing a single endpoint in the three mouse models, cannot establish a definitive relationship between duration of cachexia and severity of bone loss, as they cannot exclude that more severe cancer progression would lead to even more exacerbated bone loss. Moreover, the use of different mouse strains (CD2F1, athymic Nu/Nu, C57Bl6-Apc^Min/+^), either immunocompetent or immunodeficient, may prevent us from generalizing our results and could represent a limitation to our findings. Indeed, strain-specific bone phenotypes have been previously described in a transgenic model with either a BALC/cJ or C57Bl6/J background, as well as in athymic and euthymic mice (Mccauley et al., 1989; Syberg et al., 2012). Similarly, bone regeneration and increased risk of osteoporosis have been shown in NOD/scid-IL2R$\gamma_{\text{c}}^{\text{null}}$ animals, which exhibit defects in innate and adaptive immunity (Rapp et al., 2016), as well as in patients affected with acquired immunodeficiency syndrome (Annapoorna et al., 2004). Interestingly, the level of physical activity may also contribute to explain the loss of bone tissue across the three tumor models, especially keeping in mind that the interaction between skeletal muscle and bone tissue was initially described as mainly mechanical in nature (Brotto and Bonewald, 2015). Consistently, previous reports showed that the growth of colorectal tumors progressively affected the overall activity and physical performances in C26 hosts and Apc^Min/+^ mice (Baltgalvis et al., 2010; Toledo et al., 2014).

Nonetheless, muscle-derived factors have been shown to significantly affect bone metabolism, although it is not completely clear whether changes in muscle mass *per se* may also affect the integrity of bone tissue (Hamrick, 2012; Brotto and Bonewald, 2015). In the present study, that mainly described the relationship between colorectal cancer and the occurrence of changes in bone mechanical properties, we did not evaluate the levels of any of these factors. However, several mediators, such as BDNF, CXCL-1 (also known as KC), IL-1, IL-5, IL-6, IL-7, irisin, IFN-γ, LIF, TNF, TGF-α/β, and myostatin, have been shown to take part to the biochemical communications between skeletal muscle and bone tissue and to play a role in regulating the complicated balance between bone degradation and bone generation (Saidenberg-Kermanac'h et al., 2004; Mizoguchi et al., 2009; Polzer et al., 2010; Hamrick, 2011, 2012; Schett, 2011; Elkasrawy and Hamrick, 2013; Brotto and Bonewald, 2015; Waning et al., 2015). Importantly, bone-targeting pro-inflammatory cytokines, such as IL-6, IL-7, and IL-15, were originally described in association with muscle contraction and exercise, thus further supporting the idea that mechanical stimulation is fundamental to maintain bone integrity (Nielsen et al., 2007; Pedersen, 2009; Haugen et al., 2010; Hamrick, 2012). Conversely, recent evidence suggests that bone, acting as an endocrine organ, may secrete factors that can target muscle tissue and influence its homeostasis (Dallas et al., 2013). Indeed, elevated FGF23 was reported to affect cardiac function (Mirza et al., 2009), while osteocalcin levels were shown to have a direct effect on muscle strength (Fernández-Real et al., 2009).

Recent evidence also suggests that anticancer therapies may contribute to both muscle weakness and bone decay. Along this line, we recently showed that therapies routinely used for the treatment of colorectal cancer play an important role in promoting muscle wasting and fatigue, particularly by affecting the muscle oxidative state and causing mitochondrial depletion (Barreto et al., 2016). Of note, significant loss of BMD was described in patients undergoing adjuvant chemotherapy for various gynecologic cancers (Christensen et al., 2016; Lee et al., 2016) or radiotherapy for abdominal tumors (Wei et al., 2016). Regardless of the molecular causes responsible for these side effects, the occurrence of bone fractures in patients with cancer or undergoing chemo-radiotherapy represents a problem of significant concern, causing substantial morbidity and worsening of the quality of life.

In conclusion, the data presented in our study suggest that colorectal cancer associates with muscle wasting and is generally accompanied by bone loss (Figure 5). Based on our results, the extent of bone depletion might depend upon tumor type, burden and duration of the disease, although limitations associated with the use of different mouse strains were also identified. Despite all this, the identification of muscle-/bone-derived factors that may result into novel therapeutic targets for the treatment of sarcopenia and osteoporosis is far from being accomplished. Moreover, while it is largely accepted that strategies aimed at preserving muscle mass can improve survival and quality of life in cancer cachexia (Benny Klimek et al., 2010; Zhou et al., 2010), as well as tolerance to the anticancer therapies (Barreto et al., 2016; Hatakeyama et al., 2016), further studies will be required to clarify whether preserving bone mass in cachexia may represent a novel strategy to improve outcomes and survival in colorectal cancer.

## Author contributions

AB, LK, and TZ conceived of the studies. JC, TG, KM, AR, MC, LK, and TZ provided essential equipment, expertise and resources. AB, VP, RM, MP, KK, and RB performed experiments. AB, JK, VP, RM, TZ, KM, and AR analyzed and interpreted data. AB, JK, MC, LK, and TZ wrote and edited the manuscript.

### Conflict of interest statement

The authors declare that the research was conducted in the absence of any commercial or financial relationships that could be construed as a potential conflict of interest.
